# Protocol of an open, three-arm, individually randomized trial assessing the effect of delivering sexual and reproductive health information to young people (aged 13–24) in Kenya and Peru via mobile phones: adolescent/youth reproductive mobile access and delivery initiative for love and life outcomes (ARMADILLO) study stage 2

**DOI:** 10.1186/s12978-018-0568-6

**Published:** 2018-07-11

**Authors:** Lianne Gonsalves, Michelle J. Hindin, Angela Bayer, Cesar P. Carcamo, Peter Gichangi, Ndema Habib, Jefferson Mwaisaka, Lale Say

**Affiliations:** 1 Department of Reproductive Health and Research including UNDP/UNFPA/UNICEF/WHO/World Bank Special Programme of Research, Development and Research Training in Human Reproduction (HRP), World Health Organization, Avenue Appia 20, 1201 Geneva, Switzerland; 2 Population Council, Department of Reproductive Health, 1 Dag Hammarskjold Plaza, New York, NY 10017 USA; 3 Facultad de Salud Pública y Administración, Universidad Peruana Cayetano Heredia, Av. Honorio Delgado 430, San Martin de Porres, Lima, Peru; 4 International Centre for Reproductive Health Kenya, P.O. Box 91109-80103, Mombasa, Kenya; 5 Department of Human Anatomy, University of Nairobi, P. O. Box 30197, Nairobi, 00100 Kenya; 6 Ghent University, C. Heymanslaan 10, 9000 Ghent, Belgium

**Keywords:** Adolescent, Youth, mHealth, Intervention, Low-income countries, Middle-income countries, Sexual and reproductive health

## Abstract

**Background:**

Young people face special challenges to accessing needed sexual and reproductive health (SRH) information and support. With high penetration and access, mobile phones provide a new channel for reaching young people, but there is little evidence around the impact of digital interventions on improving health outcomes. The Adolescent/Youth Reproductive Mobile Access and Delivery Initiative for Love and Life Outcomes (ARMADILLO) study will assess the effect of an intervention providing SRH information to young people via text message on their mobile phones in Kenya and Peru. This protocol details an open, individually-randomized, three-arm trial lasting seven weeks to assess the effect of the ARMADILLO intervention on dispelling myths and misconceptions related to contraception. A secondary objective is to determine whether youth given access to SRH information via text message can accurately retain that information.

**Methods:**

Following a household enumeration, one eligible youth per household will be randomly selected for participation and randomized by computer into one of the three arms. Intervention arm participants will receive access to ARMADILLO content, control participants will receive no information, and ‘Arm 3’ participant interactions will vary by site: in Kenya, they will be alerted to various SRH domains and encouraged to learn on their own; in Peru, they will receive key content from each domain with the option to learn more if they wish. Participants will complete multiple-choice questionnaires administered by data collectors at baseline (prior to randomization), at intervention-period end (after week seven), and eight weeks after timing out of the intervention period.

**Discussion:**

Part of the Sustainable Development Goal commitment towards *ensuring healthy lives and promoting well-being for all at all ages* includes a commitment to ‘*ensuring universal access to sexual health and reproductive health-care services, including for family planning, information and education’.* If proven to be effective, interventions like ARMADILLO can bridge an important gap towards achieving universal access to SRH information and education for an otherwise difficult-to-reach group.

**Trial registration:**

This trial was retrospectively registered with the ISRCTN Registry and assigned registration number ISRCTN85156148 on 29 May, 2018.

## Resumen

**Antecedentes:**

Las personas jóvenes se enfrentan a desafíos únicos en cuanto a acceso a información y apoyo en temas de salud sexual y reproductiva (SSR). Debido a su elevada penetración y acceso, los teléfonos celulares brindan un nuevo canal para acceder a los jóvenes. Sin embargo, existe poca evidencia sobre el impacto de las intervenciones digitales en la mejora de los resultados de salud. La investigación ARMADILLO (Adolescent/Youth Reproductive Mobile Access and Delivery Initiative for Love and Life Outcomes en inglés y Uso de celulares para mejorar resultados de amor y vida en adolescentes y jóvenes en español) evaluará el efecto de una intervención que brinda información sobre SSR a jóvenes mediante mensajes de texto a sus celulares en Kenia y Perú. Este protocolo detalla un ensayo abierto de tres brazos aleatorizado a nivel individual que dura siete semanas y evalúa el efecto de la intervención ARMADILLO para disipar los mitos e ideas equivocadas relacionadas a los métodos anticonceptivos. Un objetivo secundario es determinar si los jóvenes que tienen acceso a información sobre SSR vía mensaje de texto pueden retener esa información de manera precisa.

**Métodos:**

Posterior a una enumeración de hogares, una persona joven por cada hogar será seleccionada al azar para ser participante y será randomizada por computadora en uno de los tres brazos. Los participantes del brazo intervención recibirán acceso a los contenidos de ARMADILLO, los participantes del brazo control no recibirán ninguna información, y los participantes del ‘brazo 3’ variarán según la sede: en Kenia, serán alertados sobre diversos dominios de SSR y animados a aprender más por su cuenta; en Perú, recibirán contenidos claves de cada dominio con la opción de aprender más si desean. Los participantes completarán encuestas de opción múltiple administradas por encuestadores durante la línea basal (antes de ser randomizados), al final de la intervención (después de la semana 7), y ocho semanas después de terminar la intervención.

**Discusión:**

Parte del compromiso de los Objetivos de Desarrollo Sostenible hacia garantizar una vida sana y promover el bienestar para todos en todas las edades incluye un compromiso a ‘garantizar el acceso universal a los servicios de salud sexual y reproductiva, incluidos los de planificación de la familia, información y educación’. Si se prueba su efectividad, intervenciones como ARMADILLO podrían tender un puente sobre una brecha importante hacia el logro del acceso universal a información y educación en SSR para un grupo que es difícil de alcanzar de otra manera.

**Registro del Ensayo:**

Este ensayo se registró de manera retrospectiva con el Registro ISRCTN y fue asignado el número de registro ISRCTN85156148 el 29 de mayo, 2018.

## Plain English summary

There is a high unmet need for sexual and reproductive health (SRH) information and services for young people worldwide. However, financial, cultural, social, and legal obstacles can make it difficult for youth to access the SRH resources they need. Mobile phones can provide an innovative, private way for young people to obtain quality SRH information; however, there is not a lot of evidence on how effective this strategy is. Therefore, this trial will randomize participants into three arms to determine if young people who are given access to SRH information via mobile phone (using text messages) can better dispel myths and misconceptions related to contraception and retain SRH information than those without this access.

This trial takes place in Peru and Kenya, where participants will be aged 13–17 and 18–24, respectively, and possess their own mobile phone. Participants will either be randomized into an intervention, control, or a third arm. Intervention arm participants will be able to access validated SRH messages developed by young people from their area in an earlier stage of research. Control arm participants will receive no intervention. Arm Three participants will receive either select SRH messages pushed to their phones daily (in Peru) or a weekly message instructing them to go learn about a certain SRH topic (in Kenya). The intervention period will last seven weeks in total, and all participants will be assessed before they are randomized, after they time out of the intervention (end of seven weeks), and after an additional eight weeks follow-up.

## Background

Today, there are 1.8 billion young people between the ages of 10 and 24–90% of whom live in low- and middle-income countries [1] – who are progressing through a period of life in which they will develop important health behaviors they will carry through their adult years. Young people will undergo puberty and face important decisions related to their sexual and reproductive health (SRH) and wellbeing including the possibility of becoming sexually active, married, and/or bearing children. Generally, young people are considered to be healthy; however, death related to maternal causes (abortion, maternal hemorrhage, maternal sepsis) is the number two killer of young women aged 15–19 and number one killer of young women aged 20–24, while HIV/AIDS is a top five killer of young men and women aged 20–24 [2]. An estimated 8.7 million young women aged 15 to 24 undergo unsafe abortions each year [3]. SRH challenges impact morbidity as well: unsafe sex and lack of contraception are the top two contributors to DALYs for young women aged 15–24 [4].

All populations face challenges when seeking to protect and promote their sexual and reproductive health. However, even when SRH services are available in a given community, added financial, cultural, social, or privacy barriers may prevent young people from accessing information about and utilizing them, especially if providers and communities are influenced by social or cultural norms that deem young people ineligible for access [5, 6]. As a result, in many regions of the world, young women wanting to avoid pregnancy can be up to twice as likely as adult women to have an unmet need for modern contraception [7], with data from 61 low- and middle-income countries (LMICs) estimating that 33 million women aged 15–24 have an unmet need for contraception [8].

Creating a safe space in which to seek SRH information and support is a critical component to developing the enabling environment by which young people can be empowered to access SRH services [9]. The rapid spread of mobile phones and related technology in recent years provides an interesting new channel for reaching youth. Young people appear to be especially enthusiastic adopters of digital technology as demonstrated by their comfort using and communicating over various channels on feature and smart phones [10]. Additionally, the nature of mobile phone technology itself ensures a level of discretion and privacy that would otherwise not exist when reaching young people on sensitive issues, such as their sexual and reproductive health [10]. Mobile devices can also theoretically transcend gender, marital status, and other demographic characteristics that may otherwise represent obstacles to accessing SRH information. However, there remains a dearth of evidence around the tangible impact that so-called ‘digital health’ interventions can achieve in improving health [11, 12].

In 2015, the World Health Organization’s Department of Reproductive Health and Research partnered with research partners at the International Centre for Reproductive Health –Kenya (ICRHK) in Mombasa, and the Universidad Peruana Cayetano Heredia in Lima, Peru, to initiate the Adolescent/Youth Reproductive Mobile Access and Delivery Initiative for Love and Life Outcomes (ARMADILLO) Study. The goal of ARMADILLO is to develop and evaluate an on-demand system for youth to access and receive SRH information through short message service (SMS, also known as ‘text message’). ARMADILLO was envisioned as a three-stage study: Stage 1 developed and tested the message content that would form the ARMADILLO system [13]; Stage 2 will rigorously assess the effect of ARMADILLO on knowledge for action, attitudes and self-efficacy; and Stage 3 will measure real-world uptake following roll-out of the service. We describe Stage 2, which uses an open, individually-randomized, three-arm trial to assess the effect of the ARMADILLO intervention on dispelling myths and misconceptions related to contraception.

### Study objectives

In making decisions about their sexual and reproductive health, young people fall on a spectrum of contraceptive use patterns [14, 15], which range from never having used contraception to being a current user of most effective contraceptive methods (long-acting reversible contraceptive methods).

**Fig. 1:**
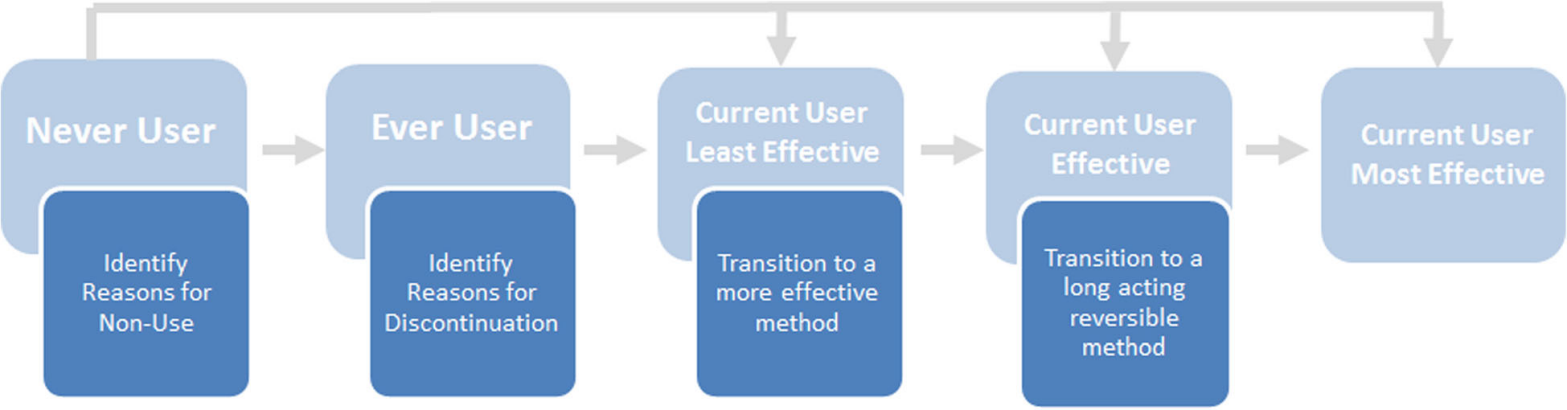
Theoretical model of contraceptive use patterns among adolescents

This spectrum (Fig. 1), is fluid, with people likely moving through multiple categories throughout their youth and reproductive years in general. At each point of transition, different strategies may be required to support individuals, should they want to avoid a pregnancy. Myths or misconceptions about contraception can delay or even prevent family planning uptake or continuation at any of these stages. Access to clear and accurate information, therefore, becomes a foundation of all strategies. ARMADILLO serves as a resource of validated SRH information for young people, using a mode of delivery that assures their privacy and comfort. Therefore, this study will assess differential learning related to SRH between those with access to a digital health intervention compared to those who are asked to learn on their own.

The primary objective of the ARMADILLO trial is to determine whether youth given access to ARMADILLO’s targeted SRH information through their mobile phones are better able to dispel contraception myths and misconceptions than those without access to ARMADILLO. The secondary objective is to determine whether youth given access to ARMADILLO’s SRH information through their mobile phones retain information on SRH longer and more accurately than those without access to ARMADILLO.

### Trial design

The study uses an individually-randomized, open three-arm comparative design lasting seven weeks. After obtaining written informed consent, individuals randomized to the ‘intervention’ arm of the ARMADILLO trial will receive access to ARMADILLO content over the course of the intervention and will use their mobile phones to access this content on demand. Those randomized to the ‘control’ arm will receive no intervention. Arm 3 will differ by site: in Kenya, those randomized to this ‘contact’ arm will be alerted to various SRH domains (e.g. relationships, pregnancy, sexually transmitted infections, etc.) and be encouraged to learn on their own; in Peru, those randomized to the ‘push’ arm will receive key messages from each domain daily, with the option to learn more if they wish.

## Methods

### Study settings

The Kenyan site for this study will be within Kwale County, one of six counties in Kenya’s former Coast province. The total population of Kwale County was projected to be 713,487 persons in 2012, with young people aged 15–29 comprising 26% of the County’s total population [16]. In 2015, contraceptive prevalence in the county was estimated to be 38.3%--noticeably lower than the national level prevalence of 53.2% [17]. The adolescent birth rate in Kwale County is also higher than the national average, with 24.2% of women aged 15–19 having begun childbearing compared to the national level of 18% [18]. Data collection will take place in Matuga constituency and Ukunda,the most populated urban area in Kwale County.

The Peruvian site for this study will be within the capital city of Lima, on the coast of Peru and home to 9.8 million people, or 31% of the population. About one-third of Peru’s adolescents live in Lima, with the highest proportions of youth living in peripheral areas similar to our study site [19]. In 2015, about one-third of Peruvian 15–19 year old females (32.1%) reported ever having sex and 13.6% of 15–19 year olds were pregnant or were already mothers [20]. The specific ARMADILLO study site will be Pampas de San Juan de Miraflores. Located in the southern cone of Lima, “Pampas” is one of the seven zones in the district of San Juan de Miraflores, which is one of Lima’s 43 districts. Pampas’ 46 settlements (pueblos jóvenes) are home to approximately 50,000 residents [21].

### Eligibility criteria

Eligibility criteria for the ARMADILLO study is as follows:

- Youth between the ages of 13–24 (age range narrowed for each site);
- Literate
- Have their own mobile phone (meaning it is primarily in their possession, and they control when and with whom they share access) and report regular use
- Have a mobile phone with them at the time of recruitment
- Report current use of text messaging

In Kenya, participants will be between 18 and 24 years old. In Peru, participants will be between 13 and 17 years old. For privacy purposes, this stage requires participants to have access to their own mobile phones; the Kenya age range reflects formative phase findings that phone ownership dropped precipitously before age 18. In Peru, where younger youth have access to their own mobile devices, the lower age range of participants is a direct response to recommendations from health- and education- sector stakeholders in Peru, who requested that the ARMADILLO study be directed to a younger age group – specifically, 13–17 year olds – to be more in line with national government programs to reduce adolescent pregnancy.

### Intervention

#### The ARMADILLO intervention

The ARMADILLO system has been describe elsewhere [13], but in brief, consists of a free, automated, menu-based and on-demand SMS platform that provides validated SRH information across a variety of youth-identified domains of interest, including puberty, relationships, sex, contraception, HIV/STIs, and rights. SMS or ‘text message’ was chosen as a mode of delivery due to the universal access of the channel, regardless of type of phone owned.

The ARMADILLO architecture was developed in each study country using national and global guidelines around youth sexual and reproductive health, with message wording and content vetted by youth themselves during the study’s formative phase. This platform is meant to catalyse additional conversations and information- and service-seeking about SRH by providing brief, essential, guidelines- based information at the moment it is needed (with the ability to save the information for future reference).

The structure of the ARMADILLO system, including how each domain is labelled, the sub-topics nested within each domain, and the language of the message itself differs by site. In Peru, ARMADILLO domains are organized as questions: ‘Who am I?’ ‘Who looks after me?’ and ‘How do I take care of myself?’ Meanwhile in Kenya, domains are thematically focused, for example: ‘Puberty and Anatomy’ and ‘Pregnancy Prevention’. Each domain has a menu of 5–10 numbered sub-topics, with a user indicating which sub-topic he/she wishes to learn more about by replying to the system with an SMS of that number. Finally, each queried sub-topic provides information through 2–3 SMS, totalling 320–480 characters. In Kenya, the system is in both English and Swahili (with users able to indicate their language preference). In Peru, the system is in Spanish.

### Study arm descriptions

#### Intervention arm

Those assigned to the intervention arm will be provided access to one new domain (SRH topic) every week (Day 1 of 7 of a given week) and with an SMS ‘quiz’ to maintain engagement at the end of that week (Day 7 of 7). These participants will have the ability to navigate freely across and between messages, accessing subdomains that interest them and ignoring those that don’t. At the start of the next week (Day 1 of 7), access to the previous week’s domain will close – meaning that the user can no longer access the information in that domain. However, any messages the user has already received will remain in his/her phone unless deleted by the user. At the same time, the next domain will be ‘unlocked’ and the user will receive a message alerting them to this new access.

Progression through the intervention will be as follows:

- Start of Week 1: an SMS alerting intervention participants to a new domain is pushed to their phones (e.g. ‘You’ve unlocked PREGNANCY. Want to learn more? Send ‘ARMADILLO’ to XXX – free and confidential! Stay tuned for a quiz at the end of the week to win free airtime!’).
- Week 1, Days 1–7: participants can access all subdomain messages within this given domain, as many times as they would like, without charge.
- Week 1 (Day 7): a close-ended SRH outcome-assessing question is pushed via SMS to the participants’ phones. Participants receive free airtime for any response (whether or not it is correct).
- Week 2, Day 1: access to Week 1 domain ends and intervention participants receive an SMS alerting them to the new domain they can now access.
- Weeks 2–7: The pattern described for Weeks 1 and 2 continues until participants have received one week of access to all domains.

### Comparison arms

#### Control arm

Some participants will be randomly allocated to the control arm. Control arm participants will emulate standard access to SRH information and so receive no messages on their phone during the intervention period. Instead, they will only participate in a baseline assessment (described above), and in endline and follow-up assessments (described below).

#### Arm 3: ‘Contact’ (Kenya), ‘push’ Peru

The third arm has been developed in close collaboration with each study site, and aims to address specific questions from countries and/or the digital health literature.

##### Kenya

In Kenya, the ‘contact’ arm will allow for assessing whether changes in SRH outcomes are attributable to the content of the intervention or to the participant-contact nature of the intervention itself. As such, the ‘contact’ arm will match the number of system-initiated contacts without providing them access to ARMADILLO content. If the success of digital health interventions is merely in participant contact (i.e. encouraging users to consider a relevant SRH topic) rather than the informational content, we would expect no differences in outcomes between intervention and contact arms, and participants in both arms would likely have better outcomes than control participants.

Therefore, participants in Kenya’s contact arm will not have access to ARMADILLO message content. They will, however, receive the same number of system-initiated contacts (pushed messages) that the intervention arm receives: specifically, they will receive messages alerting them to a new SRH domain at the beginning of each week, and the domain-specific assessment at the end of the week.

Progression through the contact arm will be as follows:

- Start of Week 1: an SMS alerting contact participants to a ‘topic of the week’ is pushed to their phone (e.g. ‘This week’s topic is PREGNANCY. Find out what you can about pregnancy, test your knowledge with our quiz at the end of the week and win free airtime!’).
- Week 1, Day 7: There is no further interaction with the contact arm until the end of the week, when a close-ended SRH outcome-assessing question is pushed via SMS to the participants’ phones. Participants receive free airtime for any response (whether or not it is correct).
- Week 2, Day 1: access to Week 1 topic ends and contact participants receive an SMS alerting them to the new topic that they can now learn about.
- Weeks 2–7: The pattern described for Weeks 1 and 2 continues until participants have received one week of suggested topics.

##### Peru

In Peru, push-message public health campaigns are the norm for a wide variety of sensitive (re: HPV vaccination) and non-sensitive (maternal health, nutrition, etc.) health campaigns. Bi-directional interventions do not exist; this will make the on-demand format of the ARMADILLO intervention arm novel. As such, Arm 3 for Peru will adopt the Peruvian ‘push system’ norm, answering a question important for adoption and scale up to national level in this context: how does free, active engagement (intervention arm) compare with passive receipt of messages (Arm 3) for engaging with users?

Progression through the push arm will emulate existing public health campaigns in Peru and will be as follows:

- Start of Week 1: an SMS alerting participants to a ‘topic of the week’ is pushed to their phone.
- Week 1, Day 2–5: 1–2 SMS messages are pushed to participants’ phones at the same time daily (evening, when participants have left school, where phones are not generally allowed), covering various subdomains from the week’s topic
- Week 1, Day 6: At the end of the 1–2 SMS messages that are pushed to their phones on Day 6, participants have an option to ‘respond to read more’. If they respond, they will immediately receive an additional maximum of 4 messages.
- Week 1, Day 7: A close-ended SRH outcome-assessing question about that week’s topic is pushed via SMS to the participants’ phones. Participants receive free airtime for any response (whether or not it is correct).
- Week 2, Day 1: access to Week 1 topic ends and push participants receive an SMS alerting them to the new topic that they will now learn about.
- Weeks 2–7: The pattern described for Weeks 1 and 2 continues until participants have received one week of suggested topics.

### Digital health intervention implementation logistics

Messages pushed to phones incur no cost for the recipient. However, the study design requires intervention and Arm 3 participants to both send and receive messages. Sending SMS does incur charges for the study participants. Therefore, both intervention arm and Arm 3 will be zero-rated, with any SMS charges reversed billed to the study. Participants, during the enrolment process and periodically during the intervention, will be reminded that any interaction with the study is free. Participants will also have access to free airtime upon responding to the SMS quiz – this airtime will be credited to users by the study team.

Participants will also be periodically reminded that they can opt out at any time by sending a ‘STOP’ command to a short code. This opt-out option helps address a lingering ethics concern that a person may choose to exclude themselves from a study because their access to messages has compromised their comfort or security. As such, should a member of either SMS-receiving arms indicate via shortcode that they wish to be unenrolled, they will be immediately and automatically unenrolled from the study.

Arm 3 and intervention participants in both sites will progress through domains sequentially, based on numbers assigned to each domain. However, participants will be randomized as to which number domain they start with, in order to account for participant recall bias at the endline assessment as a result of having seen domains at different points across the seven weeks. For example, a participant may be randomly assigned to start at Domain 3, meaning they would cycle through Domains 4–7 in the following weeks before finishing with Domains 1 and 2.

### Outcomes

All outcomes are linked to domains and content from the ARMADILLO system. The primary outcome, dispelling myths and misconceptions about contraception, will be assessed using an index of 8–10 of the most salient myths and misconceptions about contraception [22] (for example: family planning causes cancer, family planning can make a woman barren, etc.) in a given site. The index will be country-specific (though certain myths are likely to be relevant in both settings) and developed based on a review of relevant literature and a series of focus group discussions conducted in each study area prior to the start of this research. Those in the intervention arm are hypothesized to believe fewer contraception myths than those in control arm and Arm 3 and should therefore have a significantly lower index score at endline than they did at baseline, compared to the other two groups.

Secondary outcomes of interest concern change (and retention of change) in knowledge of contraception; knowledge of puberty/anatomy; knowledge of HIV/AIDS and its transmission; attitudes around engaging in sexual activity (with self and others); attitudes around intimate partner violence; attitudes around family and peer support; and previous behaviour around sex and contraception use. These concepts will be measured using scales and indices that each consist of multiple questions to comprehensively measure the concepts. These have been pulled from a number of validated survey instruments [22–28] including (but not limited to) the Guttmacher Survey of Young Adults [29] and the Demographic and Health Survey (DHS) Program [30].

### Sample size

A sample size of 705 participants per site will provide 80% power to detect a 10% change in mean number of myths believed from baseline to endline, assuming that baseline level of belief is 55% (or .55, with an assumed standard deviation of .30) [22] and accounting for a dropout rate of up to 20% in each site. The sample size is calculated to allow for pairwise comparisons between all three groups. The slight increases in sample sizes in each site are to allow for balance in sample size across site-specific age groups as well as for males and females.

### Recruitment and allocation

We will use household-based surveys and multi-stage random sampling. First, recent satellite images or a recent census will be used to identify and enumerate all of the households in the study zone. A random sample of blocks of households will then be selected. Next, the research team will carry out a census of households to enumerate all eligible (those meeting the eligibility criteria for this study) youth in each selected block.

Following the enumeration, a member of the study team in each site will randomly generate (using a computer-based random number generator) a list of potential participants to be sampled. To minimize contamination, only one youth from each household will be able to participate in this study. The list will be a random generation of 1) the households to be sampled and 2) one eligible youth to be recruited from within that household.

Data collectors (none of whom will be involved in the randomization process described above) will be sent to a specific geographic area with the list of households. Recruitment will take place over a one month period. If the youth selected is not at home (and/or, in the case of Peru, the parent/guardian is not at home), the data collector will make an appointment for a second visit. Upon visiting the household, the individual will be consented on the spot. In the case of Peru, where participants are under age 18, parent/guardian consent will also be obtained at this point. If that individual does not wish to participate, no other eligible member of the household may be substituted.

Following recruitment, consenting, and completion of the baseline survey, the data collector will leave the household and each participant will be randomized off-site in a 1:1:1 allocation ratio using a computer-based randomization tool to intervention developed using Node.js and docker. The randomization will be overseen by a research team member does not interact with participants. Once randomized, the participant’s seven-week interaction with the relevant arm will begin the following day.

### Data collection methods

#### Assessments

Across all arms, outcomes will be assessed via an in-person questionnaire, administered by a trained researcher, with data collected on a mobile phone or tablet. The questionnaires will be administered:

- At baseline (prior to a participant being randomized into one of the three arms);
- At intervention end (following seven weeks of participation in one of the three arms); and
- At eight weeks following the end of the intervention period.

All in-person study activities will take place in a private room at a location and time that is convenient for participants. Locations will allow for both visual and auditory privacy. A project staff member will explain the study activity and answer any questions prior to and/or following completion of the given study activity.

Following recruitment and consent, all participants will complete a baseline, close-ended survey with questions linked to ARMADILLO content. Participants will be told that, should they be randomized to intervention or Arm 3, they can expect their first message from the system the following day. Following completion of the baseline survey, the participant is randomized into one of the three arms.

During the last week of an individual’s intervention period (Week 7) the participant will be contacted to schedule an in-person assessment of all outcomes at the end of the intervention. Research team members will update participant contact information if necessary and alert participants that they will be invited back for a follow-up assessment in two months.

There will be no contact with participants from any study arm in the eight weeks between the end of the intervention and follow-up. The ARMADILLO system will be offline in this period as well. Two months following a participant’s end-line assessment he/she will be contacted for an in-person, follow-up assessment of all SRH outcomes. Raffles and airtime may also be used to encourage participants to return for the follow-up assessment.

Following the completion of the in-person assessment, returning participants in all arms will be provided with a short code to access the full ARMADILLO system. In the event that the system must undergo substantial revision prior to finalization, study participants will be told that they will be alerted via SMS when the final system is ready for access.

### Data management

All study results will be kept confidential by the team in either password-protected files for electronic data or locked cabinets for paper data. Only approved team members will have access to study results. A master list will be maintained that includes ID numbers that are uniquely assigned to each participant. Interview notes, consent forms, and digital files will be labelled only with these ID numbers. These master ID lists and informed consent forms will be stored together in a locked cabinet. Master ID lists and informed consent forms will be kept separately from any printed data related to the study.

All data collected will be marked with the ID number of the relevant participant – this will be the only unique identifier for any data. All hard copy documents that contain study results will be stored in a locked file cabinet (separate from the Master ID lists) that is accessible only to key study personnel. Digital data files will be stored securely on a password-protected computer and on password-protected cloud storage such as Dropbox. Study materials will be destroyed after three years. The study coordinator for each site, under guidance of the PI, will be tasked with ensuring that all files (hard and digital) have been deleted at the appropriate time. Only the study coordinator and principal investigator for each site will initially have access to the locked cabinets and password protected storage devices used in this study. If both principal investigator and study coordinator are in agreement, access to data files on cloud storage can be granted to select research staff who will be participating in the data analysis.

Any data related to the ARMADILLO system (intervention, control, and arm 3) will be stored on an instance of RapidPro, hosted by the technology partner, Ona. RapidPro, the open source communication platform of choice for this intervention, has the ability to passively track participants’ progression through various domains and message content. Data is available on what content an individual phone number accesses and how often. Tracking phone numbers also offers an unobtrusive mechanism for reducing contamination, allowing the research team to monitor the intervention to ensure that the only phone numbers accessing the content of the intervention and contact arms are those associated participants randomized to those respective arms. It should be noted, however, that within the ARMADILLO system, these phone numbers will not be linked with any identifying information on the participant. Additionally, study teams retain complete ownership of the data and of the account(s) on RapidPro. As agreed with Ona, only the study team will be able to grant access to the account to others, including Ona. Having complete ownership of the account, the study teams will be able to completely delete their own data once the project is finished.

It should be noted that while the intervention makes use of mobile network operator (MNO) infrastructure (with text messages being relayed from RapidPro, then through an aggregator, to participants’ phones), there are no special privacy or confidentiality concerns to users arising from their respective carrier. MNOs in each country will have no way of knowing the content of the messages being sent to participants. MNOs will have no more ability to monitor users who happen to be ARMADILLO participants than they would regular users.

### Statistical analysis

After assessment of the randomization of participants, if there are no differences between arms in terms of sociodemographic characteristics, differences between arms in the contraception myths index score at endline will be assessed using standard methods—comparisons of proportions (chi-square tests) and means (t-tests) between arms and difference-in-difference techniques. If the randomization was not successful, statistical adjustment with multivariable regression will be used to assess intervention impact. It is envisioned that analyses will be conducted as intent-to-treat. Separately, similar secondary analyses will be conducted on knowledge gained and attitudes shifted in other SRH domains assessed, as well as the retention of both primary and secondary outcomes over time (through the follow-up period).

### Research ethics approval

The WHO HRP Review Panel on Research Projects (RP2), comprised of a committee of external reviewers, reviewed and approved the scientific and technical content of the study (protocol ID, A65892b). We then obtained ethics review and approval from the WHO Research Ethics Review Committee (ERC). Local ethics approval was also obtained from Institutional Review Boards of the Universidad Peruana Cayetano Heredia as well as the University of Nairobi/Kenyatta National Hospital.

### Ethical considerations

Those who voluntarily consent to participate in the study will be fully informed, as part of the consent process, that the study concerns SRH topics that they may consider sensitive and that it is possible that questions in the survey or message content could make the respondents feel uncomfortable. Participants will be informed, as part of the consent process that they can choose to ignore questions or leave the study altogether at any point if they feel uncomfortable, without repercussion.

Data collectors will be the same sex of participants, in order to minimize any discomfort participants may have commenting on the subject matter. Participants will be encouraged to identify an environment in which they are comfortable speaking with the research team, and a time that suits them.

Also, there are certain features of the ARMADILLO system itself which have ethical considerations worth mentioning. First, while ARMADILLO only purports to provide cursory information on the included topics, with the idea that the user accesses a different source for in-depth information and/or to take action, there is one notable exception: gender-based violence (GBV), for which a participant may require immediate support. The messages on gender-based violence will therefore contain direct linkages to site-specific resources from which to seek immediate support. Both Peru and Kenya have existing 24-h hotlines available to victims of violence. Additionally, both study sites have GBV services in the catchment area. As such, messages in the violence module will provide the relevant hotline phone number and information on where participants can seek services if needed/desired. Prior to the start of the study, each research team will liaise with GBV support and counselling centres in their respective catchment areas to be sensitized to the services available. At the point of consenting, all participants will be provided with a discrete (e.g. credit-card-sized) card that also contains the list of relevant hotlines and GBV services in the catchment area.

Second, ARMADILLO was developed as an on-demand system to preserve the privacy of users. However, participants in this study must also receive occasional messages pushed to their phone (quizzes and topic/unlocked domain alerts), thereby sacrificing some of the privacy that a purely on-demand system would offer. In order to compensate, eligibility criteria requires that participants in this phase have their own mobile devices, addressing a concern of an SMS appearing on a shared phone when the study participant is not using it.

A common concern in digital health interventions is whether there is a privacy-related risk to participants, in that an outside person (parent/caregiver, partner, friend) may view the messages on the participant’s phone against their wishes. This was a concern first flagged during the formative stage of research and so was thoroughly explored over the course of data collection. In both sites, formative data indicates that even if partners, peers and/or parents were to come across information from the study, there would be minimal – if any – repercussions. In Kenya parents, caregivers, and stakeholders engaged with the formative phase confirmed that their communities would view mobile phones as a valuable means of providing SRH information to young people. Meanwhile, in Peru, respondents indicated that health-oriented message campaigns were common place and would be acceptable so long as they appeared as ‘public service messages’. As such, privacy concerns are anticipated to be a non-issue; however, should we find otherwise over the course of data collection, the study will not continue.

## Discussion

Part of the Sustainable Development Goal commitment towards *ensuring healthy lives and promoting well-being for all at all ages* includes a commitment to ‘*ensuring universal access to sexual health and reproductive health-care services, including for family planning, information and education…’* [31] As previously described, young populations face added challenges to accessing the SRH information and services to which they are entitled. Interventions like ARMADILLO can bridge an important gap towards achieving universal access to SRH information and education for an otherwise difficult-to-reach group.

While use of SMS to deliver information to young people is a popular strategy, it has yet to be rigorously evaluated [13]. In this regard, ARMADILLO contributes needed data: the proposed study utilizes a rigorous design, in two countries, to test youth-developed messages. Additionally, the structure of this system is such that data collection teams in each country will be able to monitor important process indicators (related to users’ progression through the system and interaction with various domains, for example) which will provide information needed to plan for a broader rollout or scale-up of the service.

This evaluation is strategically positioned to take place prior to the start of a broad rollout of the full ARMADILLO architecture and content and a corresponding assessment of the system’s coverage. Assessing the learning potential of the system before the rollout has a few important results. First, the chance for contamination between arms is reduced (there are no promotional materials or campaigns that could contaminate Arm 3 or control participants). Second, conducting the impact assessment first allows for a ‘proof of concept’ approach to the broader ARMADILLO study – prove first that the intervention can make a difference, then roll it out broadly.

## Abbreviations

ARMADILLO: Adolescent/Youth Reproductive Mobile Access and Delivery Initiative for Love and Life Outcomes
GBV: Gender-based violence
HRP: UNDP-UNFPA-UNICEF-WHO-World Bank Special Programme of Research, Development and Research Training in Human Reproduction (HRP)
MNO: Mobile network operator
RP2: WHO HRP Review Panel on Research Projects
SMS: Short message service (text message)
SRH: Sexual and reproductive health
WHO: World Health Organization
